# A homozygous *TRIP13* pathogenic variant associated with familiar oocyte arrest and prematurely condensed sperm chromosomes

**DOI:** 10.1186/s13039-025-00722-7

**Published:** 2025-07-23

**Authors:** Michal Schweiger, André Reis, Esen Gümüslü, Alice Krebsova, Andreas Raab, Christine Lang, Denise Horn, Karl Sperling, Heidemarie Neitzel

**Affiliations:** 1 Center for Molecular Medicine Cologne, Laboratory for Epigenetics and Tumour Genetics, University of Cologne, Cologne, Germany; 2 Institute of Human Genetics, Universitätsklinikum Erlangen, Friedrich-Alexander-Universität Erlangen-Nürnberg, Erlangen, Germany; 3 Department of Cardiology, Center of Inherited Cardiovascular Disorders, Institute for Clinical and Experimental Medicine (IKEM), Prague, Czech Republic; 4 ORGANOBALANCE GmbH, Berlin and Belano Medical AG, Hennigsdorf, Germany; 5 Institute of Medical and Human Genetics, Charité-Universitätsmedizin Berlin, Berlin, Germany

**Keywords:** Human TRIP13, Yeast pch2, Oocyte arrest, Premature chromosome condensation, Sister chromatid exchanges, Complementation study

## Abstract

We report on a consanguineous family with two infertile sisters with oocyte arrest and prematurely condensed sperm chromosomes. A genome-wide linkage scan and exome sequencing revealed a homozygous variant in the gene for the thyroid receptor interacting protein 13 (TRIP13), c.518G˃A (p.Arg173Gln), affecting an evolutionary highly conserved amino acid within an ATP binding motif. Just recently, compound heterozygosity for this variant was described in a Chinese proband as pathogenic, confirming that the homozygous mutation is causative for the oocyte arrest. The *TRIP13* gene and the orthologous yeast *pch2* gene are, amongst others, involved in a meiotic checkpoint control. This checkpoint defect is obviously responsible for the premature condensation of the sperm chromosomes. *TRIP13* and *pch2* are involved in meiotic recombination. To exclude that it is involved in reciprocal somatic exchanges, we analyzed the rate of sister chromatid exchanges (SCEs) in the proband´s lymphoblastoid cells. Obviously, *TRIP13* is not involved in this type of somatic recombination. Moreover, we tested whether *TRIP13* can complement the defect of the yeast *pch2* gene. Using a yeast deletion strain lacking *pch2,* we integrated plasmids containing either the yeast *pch2* or the human *TRIP13* gene, both harboring the wild-type or the mutant allele and assessed the crossingover rate between marker genes *lys2* and *leu2* as a measure of complementation. Evidence is presented that the human plasmids, unexpectedly also that with the mutation, could complement the *pch2* deficient yeast strain, underlining that the evolutionary conservation at the molecular level obviously extends to the functional level.

**Supplementary Information:**

The online version contains supplementary material available at 10.1186/s13039-025-00722-7.

## Introduction

Since the introduction of in vitro* fertilisation* (IVF) in 1978 and later of the intracytoplasmic sperm injection (ICSI) technique, more than 10 million children in the United States have been conceived via these assisted reproductive technologies [4, 39, 49]. In parallel, there is an increase of cytological and molecular genetic studies in cases of fertilization failure and early embryo arrest ([15, 20, 48]. To the best of our knowledge, the first probands with oocyte maturation arrest after IVF were published in 1986 [44]. The oocytes showed no pronuclei, but prematurely condensed sperm chromosomes of the G1-phase besides the haploid set of maternal metaphase II chromosomes. This phenomenon of premature chromosome condensation (PCC, [21]) could be explained by the arrest of the oocyte chromosomes at metaphase II after sperm penetration and the continuing presence of the active maturation promoting factor (MPF) leading to the induction of prematurely condensed sperm chromosomes [22, 24]. Experimentally, this phenomenon has been induced by injection of isolated plant nuclei into maturing Xenopus oocytes, illustrating that such fundamental biological processes like chromosome condensation in mitosis and meiosis, which are not different in animal and plant kingdom from a morphological point of view, are also very similar with respect to the underlying regulatory control [36, 53]. Recent advances in molecular genetics have greatly enhanced research into the genetic causes of oocyte/zygote/embryo maturation arrest (OZEMA), leading to the identification of more than 20 disease genes [34, 58].

In 2002, two sisters from consanguineous parents were reported, who underwent unsuccessful IVF treatment. Their fertilized oocytes showed no pronuclei and prematurely condensed sperm chromosomes. They had two sisters with two and four children and two married brothers without offspring. This constellation pointed to an autosomal recessive trait responsible for the oocyte arrest [45]. Based on this family, we performed homozygosity mapping with microsatellites and next generation sequencing to identify the underlying gene defect, the results of which we report here.

## Materials and methods

### Subjects

After informed consent, peripheral blood was obtained from the consanguineous parents and five of their six offspring. All had a normal female or male karyotype. Two female offspring underwent unsuccessful IVF treatments, two other sisters had delivered children, both brothers were married without children. Lymphoblastoid cell lines were established from the mother, the four sisters and one brother as described previously [33].

### Genetic analysis

#### Microsatellite mapping

Genetic linkage analysis with microsatellites was carried out on DNA of both parents and five of the six offspring (except one male offspring) by standard semi-automated methods using ABI Prism 377 (Applied Biosystems) and MegaBACE 1000 (Amersham Pharmacia Biotech) sequence detection systems as described before [18]. In short: The genome-wide scan included 382 polymorphic markers labeled with fluorescein and spaced at intervals of about 10 cM throughout the genome. Additional microsatellite markers were used for fine mapping. Linkage analysis was performed assuming autosomal recessive inheritance, full penetrance and a disease gene frequency of 0.0001. Multipoint lod scores were generated with GENEHUNTER, version 1.3 [26], and reconstructed haplotypes with GENEHUNTER by hand.

#### Next generation sequencing and mutation detection

Next generation sequencing was performed with the SOLiD technology (Applied Biosystem). The raw data were mapped to the haploid human reference sequence hg18 and variants were called using the platform’s standard software pipelines [41]. Called variants were filtered to exclude variants not found in all affected persons as well as common variants identified in the dbSNP130 or HapMap databases and characterized by PolyPhen-2 [1] and Mutation Taster [46].

Mutation screening in candidate genes was performed by bidirectional sequencing of PCR products using BigDye Terminator chemistry on an ABI 3730 sequencer. Detection of variants was performed with the GS Reference Mapper Version 2.0.0.12 (Roche). Only the HCDiff (high confidence differences) of the GS Mapper software were used as basis of variant detection. As additional quality criteria we used only variants with a coverage > 10 × of high-quality reads [50].

Exome sequencing of both affected probands (II.1, II.2) and their healthy sister (II.6) was performed on an Illumina NovaSeq 6000 sequencer using the Twist Human Core Exome Enrichment Technology (Twist Bioscience Inc.) and processed as previously described [7]. In total, 443 genes associated either with HPO terms “female infertility” (HP:0008222) or “infertility” (HP:0000789), “familial cancer” or “increased SCE rate” were evaluated. This latter category included *ATM* [3], *BLM* & *WRN* ([31]; [5, 32]), *BRCA1*, *BRCA2*, *RAD51* & *POLQ* [14, 16], *FANCA* [35], *TP53* [42, 51] genes. Variants were analyzed regarding their occurrence and frequencies in population or variant databases, their segregation and their consequences as well as regarding their plausibility in light of the phenotype.

#### Sister chromatid exchange (SCE) analysis

SCE rates were assessed in lymphoblastoid cultures which were grown in medium containing 5-bromo-2-deoxy-uridine for 72 h and kept in the dark. Colcemid (0.1 μg/ml) was added to each culture two hours before the end of incubation. The cells were transferred to slides and air-dried chromosome preparations were made. Slides were stained with Giemsa and scored blindly by the same reader. Fifty well differentiated diploid second division metaphases were scored of each subject [37].

#### Yeast complementation assay

The complementation analysis between the human *TRIP13* and the yeast *pch2* gene is based on the yeast BY4743 deletion strain without the *pch2* gene, kindly provided by EUROSCARF. For methodical details see [13]. As measure of complementation the crossingover rate between the marker genes *lys2* and *leu2* was used. The BY4743 strain was heterozygous for the *lys2* gene (*lys2*^+^*,lys2*^*−*^) and transformed with two variants of the *leu2* gene (*leu2*^+^*,leu2*^*−*^). The genetic distance between the *leu2* and *lys2* genes was approx. 30 cM.

Thereafter, this strain (Y33326) was complemented with plasmid p416GPD [47] containing the human *TRIP13* gene with the wild-type (TRIP13wt) or the mutant allele c.518G˃A (TRIP13mut). As control served the Y33326 strain complemented with the yeast *pch2* gene with and without intron (pch2wti; pch2wt) or with a deletion (pch2 Δ). As further control served the wild type strain with the *pch2* gene (Y20000) and also this strain transformed with the empty p416GPD vector (wt). Transformation was confirmed by Sanger sequencing and PCR of the vectors and the leu2 cassette.

For the generation of the construct p416GPD-TRIP13, a DNA fragment encoding the full-length *TRIP13* was amplified via PCR from the cDNA WI38 (ATCC®CCL-75.1) by using the oligonucleotides TRIP13_BamHI (TTACGCGGATCCATGGACGAGGCCGTG) and TRIP13_XhoI (CCGCTCGAGTCAGATGTAAGCTGCAAGC). The resultant DNA fragment was treated with the restriction enzymes BamHI and XhoI and ligated into the BanHI/XhoI sites of the yeast vector p426GPD. Underlined primer sequences represent the restriction sites. 

The quick-change XL site directed mutagenesis kit was used to create the mutant p426GPD-TRIP13_R173Q by using the mutagenesis-primer TRIP13_R173Q_FW (CATCACCTGGAACCAGGTGGTGCTGCTCC) and TRIP13_R173Q_RV (GGAGGCAGCACCACCTGGTTCCAGGTGATG) respective to the manufacturing protocol. All generated constructs were validated by Sanger sequencing.

To calculate the crossingover rate, the various strains were stimulated to sporulate. The spores were then plated on agar plates and the resulting colonies were replica plated on leucine dropout, lysine dropout and complete medium plates. Thus, the four possible genotypes, which were either prototrophic ( +) or auxotrophic (−), could be distinguished: lys^+^ leu^+^, lys^−^ leu^−^, lys^+^ leu^−^ and lys^−^ leu^+^. The first two combinations corresponded to the original allele arrangement, the latter two are the result of meiotic recombination and reflect the crossingover rate. Based on this, the genetic distance in cM has been calculated by the mapping function of Kosambi [25].

### Statistics

Statistical analyses were carried out with Fisher´s exact test (two-tailed) and the Mann–Whitney U-Test (two-tailed).

## Results and discussion

**Fig. 1:**
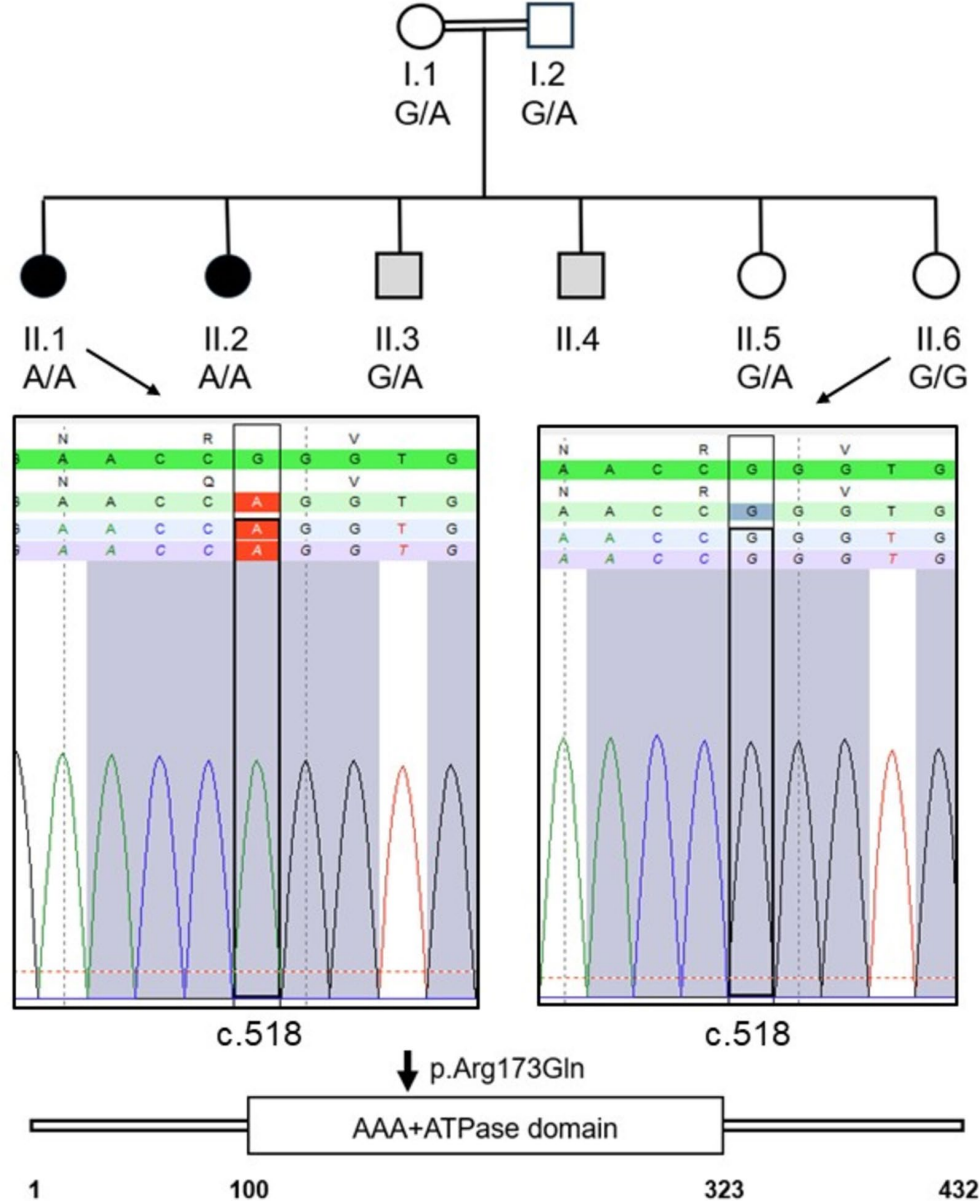
Pedigree of a consanguineous family (parents are first cousins) with two sisters with oocyte arrest (black circles), two sisters (II.5, II.6) with two and four children each and two childless married brothers (grey boxes). The oocytes of II.1 and II.2 were arrested at MI and showed prematurely condensed sperm chromosomes [45]. The affected sisters are homozygous (A/A) for the *TRIP13* missense variant (c.518G˃A) resulting in the replacement of arginine by glutamine (p.Arg173Gln). One sister (II.6) is homozygous for the wild type allele (G/G). The missense variant maps to the large AAA + ATPase domain (Ye et al. 2017)

The analysis was based on consanguineous parents with six children: Two childless married brothers, two sisters with two resp. four offspring, and two sisters who underwent unsuccessful IVF treatments (Fig. 1). After IVF their oocytes showed neither a first or second polar body nor a pronucleus, but maternal metaphase I chromosomes and prematurely condensed sperm chromosomes of the G1-phase. Both women exhibited a normal female karyotype [45]. This was a strong indication of an autosomal recessive gene defect leading to the oocyte arrest.

We carried out a genome-wide linkage scan based on 382 microsatellites evenly spaced throughout the genome. The analysis revealed five regions with homozygous markers, with only one marker on chromosomes 1 and 3 and two neighboring markers on chromosomes 5, 6 and 18 (Table 1). The most likely candidate region was the distal part of chromosome 5q with a maximal length of 21.4 cM. Two markers had produced the almost highest possible LOD score, based on the model of two affected and two unaffected sisters and heterozygote parents.

**Table 1:** Linkage analysis with microsatellite markers

chromo- some	marker	position cM	LOD score	candidate gene	map position
1	D1S419	237.2	− 1.99		
	D1S251	249	1.738	*PPP1R15B*	204,403,381–204,411,817
	D1S235	258.7	− 2		
3	D3S1262	207.2	− 99.9	none	
	D3S3663	220.3	0.931		
	D3S1265	228	− 99.9		
5	D5S2005	0	1.356	*CCDC127*	196,868–218,153
	D5S406	10.7	1.153	*TRIP13*	892,884–918,120
	D5S667	21.4	− 99.9	*ADAMTS16*	5,140,330–5,320,304
6	D6S344	1.4	− 99.9		
	D6S309	13.6	0.551	*HIST1H2BA*	25,726,909–25,727,292
	D6S422	35.7	0.997		
	D6S276	44.9	− 99.9		
18	D18S59	0.1	1.357	none	
	D18S63	7.9	0.426		
	D18S452	17.7	− 99.9		

Results of multipoint linkage analysis of seven members (excluding individual II.4) of the consanguineous family with oocyte arrest. Based on the analysis with 382 microsatellite markers five candidate regions could be identified and five candidate genes with homozygous variants identified. The physical position of the flanking markers and of the candidate genes refer to the Ensembl database, GRCh38).

After exome sequencing six genes with homozygous variants could be identified within the five candidate regions (Table 1). However, only two genes, *CCDC127* and *TRIP13*, on chromosome 5q showed potential pathogenic variants, based on the analysis with MutationTaster and PolyPhen-2. The variant c.256G˃A (p.Ala86Thr) of the *CCDC127* gene was not conserved. In contrast, the variant in the Thyroid receptor interacting protein 13 (*TRIP13*) gene, c.518G˃A (p.Arg173Gln), affects an evolutionary highly conserved position of an ATP binding motif (Fig. 1, Suppl. Figure 1a, b). There is high conservation of *TRIP13* orthologs among all eukaryotes, including yeast *pch2* [8].

The mutant mouse *trip13* gene results, amongst others, in meiotic arrest at pachytene [10, 28, 54]. Just recently, 11 *TRIP13* pathogenic missense variants and one splice variant were identified in probands with oocyte maturation arrest [9, 19, 30, 57]. One of them with oocyte MI arrest was compound heterozygous for the same *TRIP13* mutation as identified here. Thus, the homozygous variant in the probands can be considered as causative for the oocyte arrest at metaphase I.

It should be added that homozygous *TRIP13* truncating variants cause a completely different phenotype: mosaic variegated aneuploidy syndrome-3 (MVA3), characterized by chromosomal instability and early-onset Wilms tumor [55]. Moreover, amplification of *TRIP13* has been observed in various human cancers with chromosome instability, presumably due to its key role in mitotic processes, such as the spindle assembly checkpoint and DNA repair pathways [29]. The individuals with oocyte arrest and *TRIP13* missense variants including these probands, manifest only infertility without any other abnormality, including Wilms tumor.

The repair of sister chromatid exchanges (SCEs) is utilized during meiosis but difficult to assess [6]. SCEs can easily be identified in mitotic cells and are involved in DNA repair processes, such as homologous recombination [16]. Their frequency is increased after exposure to genotoxic agents and in case of pathological conditions such as Bloom syndrome [12] or Behçcet´s disease [23]. To exclude that *TRIP13* is involved in this type of reciprocal exchanges, we analyzed the SCE-rate in the proband´s lymphoblastoid cells.

**Fig. 2:**
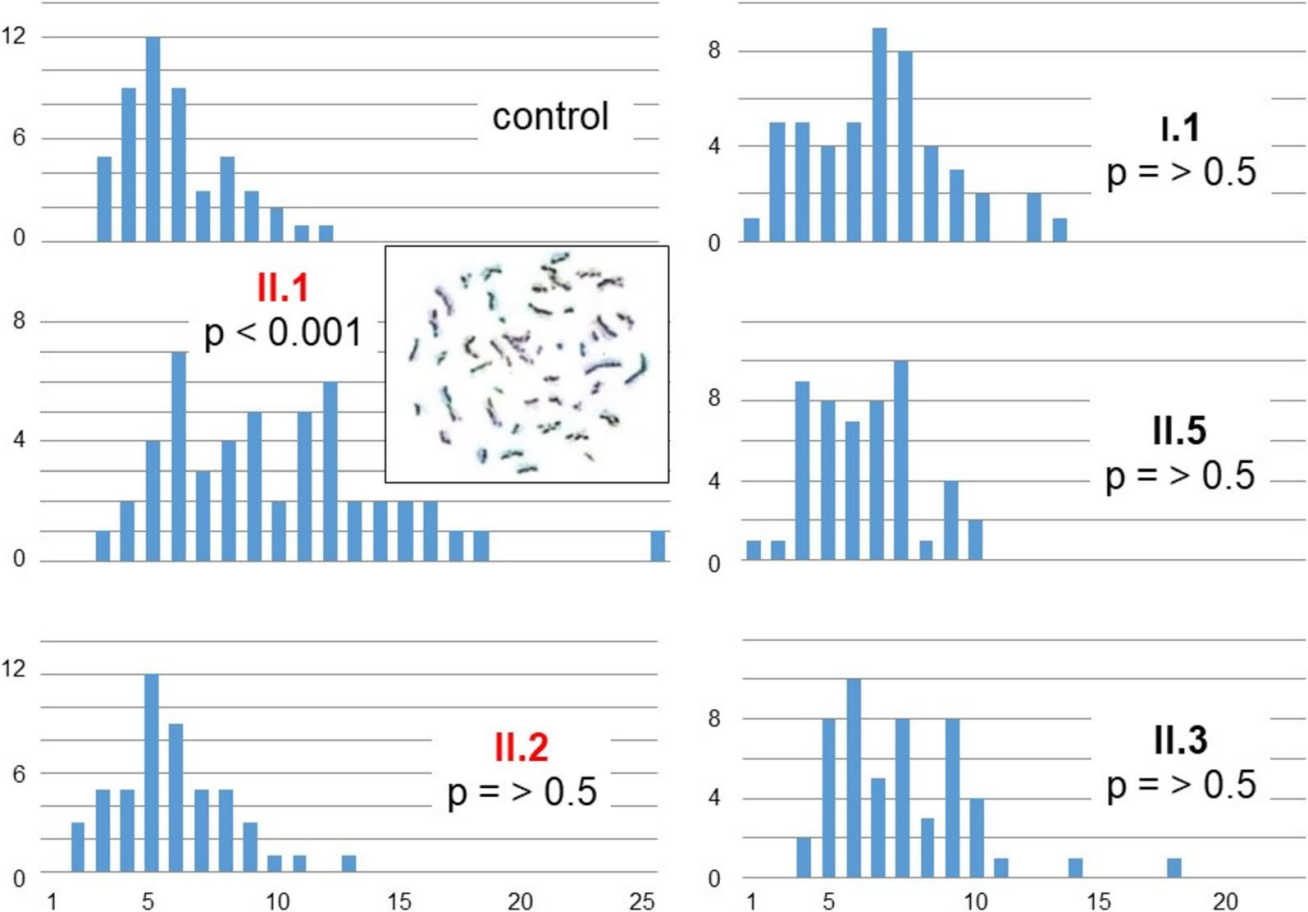
Distribution of the number of sister chromatid exchanges/cell in lymphoblastoid cells of five family members (II.1 and II.2 with oocyte arrest) and a control. The difference to the control was calculated with the Mann–Whitney U-Test, two-tailed. Inset: Metaphase of II.1 with high SCE rate

In comparison with the control there was a significant increase of the SCE rate in the affected proband II.1 (inset Fig. 2), but not in her affected sister II.2 or the heterozygote family members (Fig. 2, Suppl. Table 1). At the time of blood sampling the probands showed no signs of a viral infection and did not report any exposure to chemicals or taking drugs. Therefore, exome sequencing of both affected probands (II.1, II.2) and their healthy sister (II.6) was performed to look for a genetic disposition. In total, 443 genes associated either with infertility, familial cancer or increased SCE rate were evaluated. The observed variants were analysed regarding their occurrence and frequencies in population or variant databases, their segregation and their consequences as well as regarding their plausibility in light of the phenotype. In the healthy sister (II.6) and the affected sister with a normal SCE-rate (II.2) no relevant variants were detected, except the homozygous *TRP13* mutation in the latter (Suppl. Table 2). The affected sister with an increased SCE-rate (II.1) had a variant of the *NR5A1* gene (chr9:124493082C > A, exon 5: c.938G > A (p.Arg313His) in 20% of the reads. Therefore, mosaicism as a result of a mutation in the lymphoblastoid cell line cannot be excluded. The two sisters, II.2 and II.6 were homozygous for the wild-type allele. Thus, an influence of this heterozygote variant on the SCE-rate is highly unlikely.

In addition, the *RNF212* gene is homozygous for a Deletion-Insertion variant in exon 4: c.720_721delinsGGCTGGCTCCAGCCTGGGCAG; p.R240_S241delinsGWLQPGQ. This variant was classified as “likely benign”. RNF212 encodes a RING finger protein that is involved in meiotic recombination [38]. A homozygous *RNF212* variant, c.111dupT, resulted in nonobstructive azoospermia due to complete metaphase arrest at the spermatocyte stage [40]. Thus, an influence of this variant on the SCE rate is rather unlikely but cannot be ruled out in principle and only be clarified by further investigations. Altogether, we have no explanation for the higher SCE rate in II.1. The normal values of the other affected sister II.2 underline that this is obviously not related to the *TRIP13* pathogenic variant. Obviously, recombination mechanisms leading to meiotic crossingovers and somatic SCEs are regulated differently [52].

**Fig. 3:**
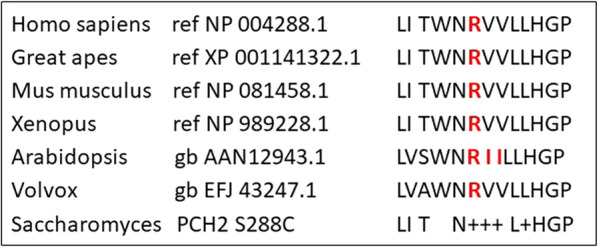
Evolutionary conservation in orthologues of selected species of the amino acid sequence flanking arginine (R) at position 173 of the human *TRIP13* gene. The yeast (Pch2) amino acid sequence is based on the Needleman-Wunsch alignment (Altschul 1997) with the human TRIP13 protein

Moreover, we tested whether the human *TRIP13* can complement a deficiency of the orthologous yeast *pch2* gene. Based on the Needleman-Wunsch global alignment algorithm [2] the identity of the *TRIP13* and *pch2* gene is 47% at the cDNA and 26% at the amino acid level. Arginine is highly conserved at position 173, but not in the relevant domain of the yeast *pch2* gene (Fig. 3). Based on the AlphaFold protein structure this region maps to a less regular and more open conformation (Suppl. Figure 2).

**Fig. 4:**
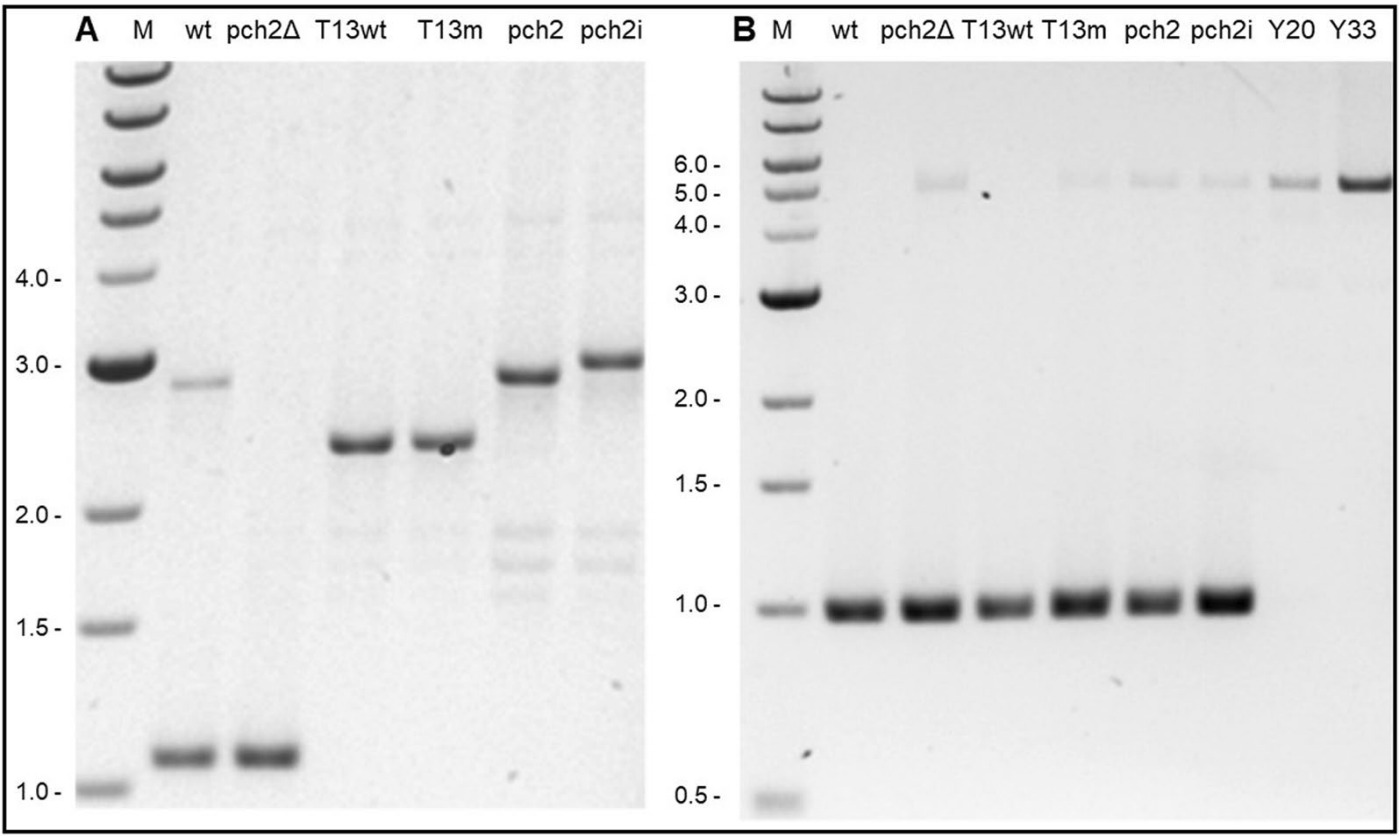
Confirmation of successful transformation of the yeast deletion strain Y33326 with the various plasmids (**A**) or the leu2 cassette (**B**). Total DNA was extracted from the various strains, the plasmids (with the genes) amplified, the PCR products separated on agarose gels and identified by UV light. The length of the bands (M) is given in kb. Wt: wild type strain Y20000 with the *pch2* gene and the empty p416GPD vector. pch2Δ: deletion strain Y33326 without the *pch2* gene, with the *TRIP13* wild type gene (T13wt) or its mutant allele c.518G˃A (T13m), with the *pch2* gene (pch2) and the *pch2* gene plus intron (pch2i). Y20: wild type strain Y20000; Y33 deletion strain Y33326

Both genes are involved in meiotic recombination. Therefore, the crossingover rate between the marker genes *lys2* and *leu2* was used as measure of complementation in the yeast Y33326 deletion strain, which contains no *pch2* gene. This strain was heterozygous for the *lys2* gene (*lys2*^+^*,lys2*^*−*^) and transformed with two variants of the *leu2* gene (*leu2*^+^*,leu2*^*−*^). The genetic distance between the *leu2* and *lys2* genes is approx. 30 cM. This strain served as one control (Y33326). Thereafter, this strain was complemented with plasmid p416GPD containing the human *TRIP13* gene with the wild-type (TRIP13wt) or the mutant allele c.518G˃A (TRIP13mut). As control, we used the complementation with the yeast *pch2 gene* with and without intron (pch2wti; pch2wt) or with the deletion mutant (pch2Δ). The wild type strain (Y20000) with the *pch2* gene and also transformed with the empty p416GPD vector (wt) was used as an additional positive control. Transformation was confirmed by Sanger sequencing and PCR of the vectors and the leu2 cassette (Fig. 4). The different yeast strains are summarized once again in Table 2.

**Table 2:** Brief characterization of the various yeast stains

	Strain	Symbol
1	Yeast wild type stain	Y2000
2	**⁄ ⁄** + empty vector	wt
3	Yeast deletion strain without the *pch2* gene	Y33326
4	**⁄ ⁄** + vector with human TRIP13 gene (wild type)	TRIP13wt
5	**⁄ ⁄** + vector with human TRIP13 gene (mutated)	TRIP13mut
6	**⁄ ⁄** + vector with yeast pch2 gene	pch2
7	**⁄ ⁄** + vector with yeast pch2 gene + intron	pch2i
8	**⁄ ⁄** + vector with yeast pch2 gene with deletion	pch2Δ

To calculate the crossingover rate, the various strains were stimulated to sporulate. The spores were then plated on agar plates and the resulting colonies were replica plated on leucine dropout, lysine dropout and complete medium plates. Thus, the four possible genotypes, which were either prototrophic ( +) or auxotrophic (−), could be distinguished: lys^+^ leu^+^, lys^−^ leu^−^, lys^+^ leu^−^ and lys^−^ leu^+^. The first two combinations corresponded to the original allele arrangement, the latter two are the result of meiotic recombination and reflect the crossingover rate (Table 3). The results illustrate that the recombination rates of the various strains containing the yeast *pch2* gene are very similar between 26.3 and 28.8%, corresponding to a genetic distance between *lys2* and *leu2* of 29–32 cM.

**Table 3:** Analysis of the crossingover rate between the various yeast strains. Random spore analysis (number of colonies) to calculate the crossingover rate between the *lys2* and *leu2* genes of the various yeast strains. P1 and P2 represent the original allele arrangement, R1 and R2 the recombinants. RF = fraction of recombinants in %. The genetic distance (cM) has been calculated by the mapping function of Kosambi [25]. Y20000: wild type strain with all yeast genes, including *pch2*; Y33326: deletion strain without the *pch2* gene. Wt: wild-type strain Y20000 with the *pch2* gene and the empty p416GPD vector. Pch2: deletion strain Y33326 with *the pch2* gene and with the *pch2* gene plus intron (pch2i) and without *the pch2* gene (pch2Δ). The deletion strain Y33326 with the *TRIP13* wild type gene (TRIP13wt) or its mutant allele c.518G˃A (TRIP13mut)

strain	Colonies	P1 lys^+^ leu^+^	P2 lys^−^ leu^−^	R1 lys^+^ leu^−^	R2 lys^−^ leu^+^	RF %	Distance (cM)	P value #	crossing-over rate
Y20000	216	71	87	26	32	26,9	30.0	–	normal
Y33326	229	81	54	45	49	41,0	57.99	0.03	⁎
wt	642	197	262	90	93	28.5	32.38	0.81	normal
pch2	640	231	241	72	96	26.3	29.16	0.90	normal
pch2i	618	233	210	91	84	28.3	32.11	0.80	normal
pch2Δ	603	137	219	108	139	41.0	57.72	0.01	⁎
TRIP13wt	317	113	73	64	67	41.3	58.83	0.02	⁎
TRIP13mut	671	305	193	84	89	25.8	28.52	0.90	normal

^#^comparison with control strain Y20000 (Fisher´s exact test, two-tailed); ⁎ significant increase of the recombination frequency (crossingover rate)

For the statistical analysis, the mean values of the spore colonies with the original arrangement of the alleles (P1/P2) and the recombinants (R1/R2) were compared between the wild type strain Y2000 and the other constructs (Table 3). The strains without the *pch2* gene showed a significant increase in the crossingover rate, which is characteristic for *pch2* mutants [56].

The results with the *TRIP13* gene were rather unexpected: the mutant was able to complement the defective yeast gene, but not the *TRIP13* wild-type gene. Moreover, the *TRIP13* findings differ from the other complementation studies in another respect. Theoretically, the ratio of P1–P2 and R1–R2 should be 1:1. This is true for the revertant in all cases, but with regard to the *TRIP13* findings, the number of P1 spore colonies was significantly higher than the P2 ones (Table 3, Suppl. Table 3). Apparently, the spores can grow better on the prototrophic medium (Lys^+^ Leu^+^) than on the auxotrophic medium (Lys^−^ Leu^−^). If one assumes that the higher P1 value reflects the situation more adequately than the P2 or the average value, the difference to the control for the construct with the *TRIP13* wild-type gene is no longer significant (*p* = 0.09, Suppl. Table 4).

Based on the observation that the codon usage between human and yeast is different, codon usage in yeast spores is not optimal for vectors with human cDNA [13]. Consequently, expression of the human TRIP13 protein is reduced, which might affect the spore growth on auxotrophic medium. The sequence alignment (Fig. 3) illustrates that no equivalent yeast amino acid exists at position 173 of the human TRIP13 protein. Thus, one cannot exclude that TRIP13 with the mutant glutamine at this position can complement Pch2 slightly better than TRIP13 with arginine. Apart from this, our results provide compelling evidence that the human TRIP13 protein can complement the crossingover defect in pch2 deficient yeast strains.

This is not unexpected as we had earlier shown that the sterol reductase deficiency in yeast with an *ERG24* mutation could be rescued by the human wildtype *LBR* gene [11] and the deletion of the x*rs2* gene in yeast complemented by the human *NBN* gene, but only with codon optimized cDNA [13]. Altogether, most of the human housekeeping genes, including those involved in mitosis and meiosis, are evolutionary conserved and even possess orthologs in unicellular plants and fungi, such as Saccharomyces cerevisiae [27]. Interestingly, a study in yeast has shown that genes involved in meiosis are characterized by positive selection and consequently more rapid evolution [43]. This might explain the rather low sequence similarity between human *TRIP13* and yeast *pch2*. 

## Conclusion

The clinical effect, infertility, of the homozygous *TRIP13* missense variant described here, is obviously limited to meiosis. This is quite remarkable if one considers, in how many tissues the gene is expressed, how diverse the clinical manifestations of *TRIP13* pathogenic variants in animals are [28] and the important role its somatic mutations play in cancerogenesis [29, 55]. On the other hand, there are many examples in which the clinical manifestation of different allelic variants can even range from mild to lethal, e.g. in the case of mutations in the *LBR* gene [11, 18], which shows similarly high evolutionary conservation as *TRIP13* and is also involved in meiotic checkpoint control [8, 17, 28]. In the present case, the oocytes are arrested in the first meiotic metaphase. The prematurely condensed sperm chromosomes indicate that this defective checkpoint is responsible for this phenomenon. There is no evidence that this is reversible and therefore all approaches to correct the oocyte arrest must be performed before fertilization [57].

## Supplementary Information

Supplementary material 1

Supplementary material 2

Supplementary material 3

Supplementary material 4

## Data Availability

The data that support the findings of this study are available in this article. The additional data sets generated (i.e. Solid sequencing, Microsatellite analysis, Exome sequencing) are available from the corresponding author on reasonable request.

## References

[1] Adzhubei IA, Schmidt S, Peshkin L, Ramensky VE, Gerasimova A, Bork P, Kondrashov AS, Sunyaev SR. A method and server for predicting damaging missense mutations. Nat Methods. 2010;7:248–9. 10.1038/nmeth0410-248. 20354512 10.1038/nmeth0410-248 PMC2855889

[2] Altschul SF, Madden TL, Schäffer AA, Zhang J, Zhang Z, Miller W, Lipman DJ. Gapped BLAST and PSI-BLAST: a new generation of protein database search programs. Nucleic Acids Res. 1997;25:3389–402. 10.1093/nar/25.17.3389. 9254694 10.1093/nar/25.17.3389 PMC146917

[3] Balmus G, Pilger D, Coates J, et al. ATM orchestrates the DNA-damage response to counter toxic non-homologous end-joining at broken replication forks. Nat Commun. 2019;10:87. 10.1038/s41467-018-07729-2. 30622252 10.1038/s41467-018-07729-2 PMC6325118

[4] Beilby KH, Kneebone E, Roseboom TJ, van Marrewijk IM, Thompson JG, Norman RJ, Robker RL, Mol BWJ, Wang R. Offspring physiology following the use of IVM, IVF and ICSI: a systematic review and meta-analysis of animal studies. Hum Reprod Update. 2023;29:272–90. 10.1093/humupd/dmac043. 36611003 10.1093/humupd/dmac043 PMC10152177

[5] Ben Salah G, Hadj Salem I, Masmoudi A, Kallabi F, Turki H, Fakhfakh F, Ayadi H, Kamoun H. A novel frameshift mutation in BLM gene associated with high sister chromatid exchanges (SCE) in heterozygous family members. Mol Biol Rep. 2014;41:7373–80. 10.1007/s11033-014-3624-5. 25129257 10.1007/s11033-014-3624-5

[6] Billmyre KK, Hughes SE. Meiosis: the elusive sister chromatid repair. Curr Biol. 2021;31:R454–6. 10.1016/j.cub.2021.03.093. 33974876 10.1016/j.cub.2021.03.093

[7] Bosch E, Hebebrand M, Popp B, Penger T, Behring B, Cox H, et al. BDV syndrome: an emerging syndrome with profound obesity and neurodevelopmental delay resembling Prader-Willi syndrome. J Clin Endocrinol Metab. 2021;106:3413–27. 10.1210/clinem/dgab592. 34383079 10.1210/clinem/dgab592

[8] Cardoso da Silva R, Vader G. Getting there: understanding the chromosomal recruitment of the AAA+ ATPase Pch2/TRIP13 during meiosis. Curr Genet. 2021;67:553–65. 10.1007/s00294-021-01166-3. 33712914 10.1007/s00294-021-01166-3 PMC8254700

[9] Chen J, Liu Y, Wu X, Zhang Y, Huang W, Han W, Chen G, Xu Q, Chen H, Wu Q, Wang J, Huang J. Identification of a novel splicing variant of thyroid hormone receptor interaction protein 13 (TRIP13) in female infertility characterized by oocyte maturation arrest. J Assist Reprod Genet. 2024;41:2777–85. 10.1007/s10815-024-03219-1. 39297991 10.1007/s10815-024-03219-1 PMC11535116

[10] Chotiner JY, Leu NA, Yang F, Cossu IG, Guan Y, Lin H, Wang PJ (2023) TRIP13 localizes to synapsed chromosomes and functions as a dosage-sensitive regulator of meiosis. bioRxiv [Preprint] Dec 15:2023.09.25.559355. 10.1101/2023.09.25.559355 10.7554/eLife.92195 PMC11361706 39207914

[11] Clayton P, Fischer B, Mann A, Mansour S, Rossier E, Veen M, Lang C, Baasanjav S, Kieslich M, Brossuleit K, Gravemann S, Schnipper N, Karbasyian M, Demuth I, Zwerger M, Vaya A, Utermann G, Mundlos S, Stricker S, Sperling K, Hoffmann K. Mutations causing Greenberg dysplasia but not Pelger anomaly uncouple enzymatic from structural functions of a nuclear membrane protein. Nucleus (Paris). 2010;1:354–66. 10.4161/nucl.1.4.12435. 10.4161/nucl.1.4.12435 PMC3027044 21327084

[12] Cunniff C, Bassetti JA, Ellis NA. Bloom’s syndrome: clinical spectrum, molecular pathogenesis, and cancer predisposition. Mol Syndromol. 2017;8:4–23. 10.1159/000452082. 28232778 10.1159/000452082 PMC5260600

[13] Demuth I, Krebs SK, Dutrannoy V, Linke C, Krobitsch S, Varon R, Lang C, Raab A, Sperling K, Digweed M. Yeast XRS2 and human NBN gene: experimental evidence for homology using codon optimized cDNA. PLoS ONE. 2018;13: e0207315. 10.1371/journal.pone.0207315. 30440001 10.1371/journal.pone.0207315 PMC6237358

[14] De Pascalis I, Pilato B, Mazzotta A, Dell’Endice TS, Rubini V, Simone G, Mangia A. Sister chromatid exchange: a possible approach to characterize familial breast cancer patients. Oncol Rep. 2015;33:930–4. 10.3892/or.2014.3628. 25434423 10.3892/or.2014.3628

[15] Hatırnaz Ş, Hatırnaz ES, Ellibeş Kaya A, Hatırnaz K, Soyer Çalışkan C, Sezer Ö, Dokuzeylül Güngor N, Demirel C, Baltacı V, Tan S, Dahan M. Oocyte maturation abnormalities - A systematic review of the evidence and mechanisms in a rare but difficult to manage fertility pheneomina. Turk J Obstet Gynecol. 2022;19:60–80. 10.4274/tjod.galenos.2022.76329. 35343221 10.4274/tjod.galenos.2022.76329 PMC8966321

[16] Heijink AM, Stok C, Porubsky D, Manolika EM, de Kanter JK, Kok YP, Everts M, de Boer HR, Audrey A, Bakker FJ, Wierenga E, Tijsterman M, Guryev V, Spierings DCJ, Knipscheer P, van Boxtel R, Ray Chaudhuri A, Lansdorp PM, van Vugt MATM. Sister chromatid exchanges induced by perturbed replication can form independently of BRCA1, BRCA2 and RAD51. Nat Commun. 2022;13:6722. 10.1038/s41467-022-34519-8. 36344511 10.1038/s41467-022-34519-8 PMC9640580

[17] Herruzo E, Lago-Maciel A, Baztán S, Santos B, Carballo JA, San-Segundo PA. Pch2 orchestrates the meiotic recombination checkpoint from the cytoplasm. PLoS Genet. 2021;17: e1009560. 10.1371/journal.pgen.1009560. 34260586 10.1371/journal.pgen.1009560 PMC8312941

[18] Hoffmann K, Dreger CK, Olins AL, Olins DE, Shultz LD, Lucke B, Karl H, Kaps R, Müller D, Vayá A, Aznar J, Ware RE, Sotelo Cruz N, Lindner TH, Herrmann H, Reis A, Sperling K. Mutations in the gene encoding the lamin B receptor produce an altered nuclear morphology in granulocytes (Pelger-Huët anomaly). Nat Genet. 2002;31:410–4. 10.1038/ng925. 12118250 10.1038/ng925

[19] Hu H, Zhang S, Guo J, Meng F, Chen X, Gong F, Lu G, Zheng W, Lin G. Identification of novel variants of thyroid hormone receptor interaction protein 13 that cause female infertility characterized by zygotic cleavage failure. Front Physiol. 2022;13: 899149. 10.3389/fphys.2022.899149. 35812326 10.3389/fphys.2022.899149 PMC9259851

[20] Huo M, Zhang Y, Shi S, Shi H, Liu Y, Zhang L, Wang Y, Niu W. Gene spectrum and clinical traits of nine patients with oocyte maturation arrest. Front Genet. 2022;13: 772143. 35140748 10.3389/fgene.2022.772143 PMC8819080

[21] Johnson RT, Rao PN. Mammalian cell fusion: induction of premature chromosome condensation in interphase nuclei. Nature. 1970;226:717–22. 10.1038/226717a0. 5443247 10.1038/226717a0

[22] Jones KT. Turning it on and off: M-phase promoting factor during meiotic maturation and fertilization. Mol Hum Reprod. 2004;10:1–5. 10.1093/molehr/gah009. 14665700 10.1093/molehr/gah009

[23] Karaman A, Kadi M, Kara F. Sister chromatid exchange and micronucleus studies in patients with Behçet’s disease. J Cutan Pathol. 2009;36:831–7. 10.1111/j.1600-0560.2008.01180.x. 19159395 10.1111/j.1600-0560.2008.01180.x

[24] Kawahara M, Wakai T, Yamanaka K, Kobayashi J, Sugimura S, Shimizu T, Matsumoto H, Kim JH, Sasada H, Sato E. Caffeine promotes premature chromosome condensation formation and in vitro development in porcine reconstructed embryos via a high level of maturation promoting factor activity during nuclear transfer. Reproduction. 2005;130:351–7. 10.1530/rep.1.00644. 16123242 10.1530/rep.1.00644

[25] Kosambi DD. The estimation of map distances from recombination values. Ann Eugen. 1943;12:172–5. 10.1111/j.1469-1809.1943.tb02321.x.

[26] Kruglyak L, Daly MJ, Reeve-Daly MP, Lander ES. Parametric and nonparametric linkage analysis: a unified multipoint approach. Am J Hum Genet. 1996;58:1347–63 (**PMID: 8651312**). 8651312 PMC1915045

[27] Lee CE, Singleton KS, Wallin M, Faundez V. Rare genetic diseases: nature’s experiments on human development. iScience. 2020;23: 101123. 10.1016/j.isci.2020.101123. 32422592 10.1016/j.isci.2020.101123 PMC7229282

[28] Li XC, Schimenti JC. Mouse pachytene checkpoint 2 (trip13) is required for completing meiotic recombination but not synapsis. PLoS Genet. 2007;3: e130. 10.1371/journal.pgen.0030130. 17696610 10.1371/journal.pgen.0030130 PMC1941754

[29] Lu S, Qian J, Guo M, Gu C, Yang Y. Insights into a crucial role of TRIP13 in human cancer. Comput Struct Biotechnol J. 2019;17:854–61. 10.1016/j.csbj.2019.06.005. 31321001 10.1016/j.csbj.2019.06.005 PMC6612527

[30] Lv XJ, Guo J, Lin G. Novel mutations in *TRIP13* lead to female infertility with oocyte maturation arrest. Yi Chuan. 2023;45:514–25. 10.16288/j.yczz.23-022. 37340965 10.16288/j.yczz.23-022

[31] Mendez-Bermudez A, Hidalgo-Bravo A, Cotton VE, Gravani A, Jeyapalan JN, Royle NJ. The roles of WRN and BLM recQ helicases in the alternative lengthening of telomeres. Nucleic Acids Res. 2012;40:10809–20. 10.1093/nar/gks862. 22989712 10.1093/nar/gks862 PMC3510502

[32] Mukherjee S, Sinha D, Bhattacharya S, Srinivasan K, Abdisalaam S, Asaithamby A. Werner syndrome protein and DNA replication. Int J Mol Sci. 2018;19: 3442. 10.3390/ijms19113442. 30400178 10.3390/ijms19113442 PMC6274846

[33] Neitzel H. A routine method for the establishment of permanent growing lymphoblastoid cell lines. Hum Genet. 1986;73:320–6. 10.1007/BF00279094. 3017841 10.1007/BF00279094

[34] OMIM (2023): OZEMA1 - OZEMA21. https://www.omim.org

[35] Palovcak A, Liu W, Yuan F, Zhang Y. Maintenance of genome stability by Fanconi anemia proteins. Cell Biosci. 2017;7:8. 10.1186/s13578-016-0134-2. 28239445 10.1186/s13578-016-0134-2 PMC5320776

[36] Pei Z, Deng K, Xu C, Zhang S. The molecular regulatory mechanisms of meiotic arrest and resumption in oocyte development and maturation. Reprod Biol Endocrinol. 2023;21:90. 10.1186/s12958-023-01143-0. 37784186 10.1186/s12958-023-01143-0 PMC10544615

[37] Perry P, Wolff S. New Giemsa method for the differential staining of sister chromatids. Nature. 1974;251:156–8. 10.1038/251156a0. 4138930 10.1038/251156a0

[38] Reynolds A, Qiao H, Yang Y, Chen JK, Jackson N, Biswas K, Holloway JK, Baudat F, de Massy B, Wang J, Höög C, Cohen PE, Hunter N. RNF212 is a dosage-sensitive regulator of crossing-over during mammalian meiosis. Nat Genet. 2013;45:269–78. 10.1038/ng.2541. 23396135 10.1038/ng.2541 PMC4245152

[39] Ribeiro S, Sousa M. In vitro fertilisation and intracytoplasmic sperm injection predictive factors: a review of the effect of female age, ovarian reserve, male age, and male factor on IVF/ICSI treatment outcomes. JBRA Assist Reprod. 2023;27:97–111. 10.5935/1518-0557.20220000. 35916467 10.5935/1518-0557.20220000 PMC10065784

[40] Riera-Escamilla A, Enguita-Marruedo A, Moreno-Mendoza D, Chianese C, Sleddens-Linkels E, Contini E, Benelli M, Natali A, Colpi GM, Ruiz-Castañé E, Maggi M, Baarends WM, Krausz C. Sequencing of a “mouse azoospermia” gene panel in azoospermic men: identification of RNF212 and STAG3 mutations as novel genetic causes of meiotic arrest. Hum Reprod. 2019;34:978–88. 10.1093/humrep/dez042. 31125047 10.1093/humrep/dez042

[41] Rödelsperger C, Krawitz P, Bauer S, Hecht J, Bigham AW, Bamshad M, de Condor BJ, Schweiger MR, Robinson PN. Identity-by-descent filtering of exome sequence data for disease-gene identification in autosomal recessive disorders. Bioinformatics. 2011;27:829–36. 10.1093/bioinformatics/btr022. 21278187 10.1093/bioinformatics/btr022 PMC3051326

[42] Roy S, Tomaszowski KH, Luzwick JW, Park S, Li J, Murphy M, Schlacher K. p53 orchestrates DNA replication restart homeostasis by suppressing mutagenic RAD52 and POLθ pathways. Elife. 2018;7: e31723. 10.7554/eLife.31723. 29334356 10.7554/eLife.31723 PMC5832412

[43] Sawyer SL, Malik HS. Positive selection of yeast nonhomologous end-joining genes and a retrotransposon conflict hypothesis. Proc Natl Acad Sci USA. 2006;103:17614–21769. 10.1073/pnas.0605468103. 17101967 10.1073/pnas.0605468103 PMC1693795

[44] Schmiady H, Sperling K, Kentenich H, Stauber M. Prematurely condensed human sperm chromosomes after in vitro fertilization (IVF). Hum Genet. 1986;74:441–3. 10.1007/BF00280502. 3793107 10.1007/BF00280502

[45] Schmiady H, Neitzel H. Arrest of human oocytes during meiosis I in two sisters of consanguineous parents: first evidence for an autosomal recessive trait in human infertility: case report. Hum Reprod. 2002;17:2556–9. 10.1093/humrep/17.10.2556. 12351528 10.1093/humrep/17.10.2556

[46] Schwarz JM, Rödelsperger C, Schuelke M, Seelow D. Mutationtaster evaluates disease-causing potential of sequence alterations. Nat Methods. 2010;7:575–6. 10.1038/nmeth0810-575. 20676075 10.1038/nmeth0810-575

[47] Sikorski RS, Hieter P. A system of shuttle vectors and yeast host strains designed for efficient manipulation of DNA in *Saccharomyces cerevisiae*. Genetics. 1989;122:19–27. 10.1093/genetics/122. 2659436 10.1093/genetics/122.1.19 PMC1203683

[48] Solovova OA, Chernykh VB. Genetics of oocyte maturation defects and early embryo development arrest. Genes (Basel). 2022;13(11): 1920. 10.3390/genes13111920. 36360157 10.3390/genes13111920 PMC9689903

[49] Sullivan-Pyke CS, Senapati S, Mainigi MA, Barnhart KT. In vitro fertilization and adverse obstetric and perinatal outcomes. Semin Perinatol. 2017;41:345–53. 10.1053/j.semperi.2017.07.001. 28818301 10.1053/j.semperi.2017.07.001 PMC5951714

[50] Timmermann B, Kerick M, Roehr C, Fischer A, Isau M, Boerno ST, Wunderlich A, Barmeyer C, Seemann P, Koenig J, Lappe M, Kuss AW, Garshasbi M, Bertram L, Trappe K, Werber M, Herrmann BG, Zatloukal K, Lehrach H, Schweiger MR. Somatic mutation profiles of MSI and MSS colorectal cancer identified by whole exome next generation sequencing and bioinformatics analysis. PLoS ONE. 2010;5: e15661. 10.1371/journal.pone.0015661. 21203531 10.1371/journal.pone.0015661 PMC3008745

[51] Tiwari A, Addis Jones O, Chan KL. 53BP1 can limit sister-chromatid rupture and rearrangements driven by a distinct ultrafine DNA bridging-breakage process. Nat Commun. 2018;9:677. 10.1038/s41467-018-03098-y. 29445165 10.1038/s41467-018-03098-y PMC5813243

[52] van Heemst D, Heyting C. Sister chromatid cohesion and recombination in meiosis. Chromosoma. 2000;109:10–26. 10.1007/s004120050408. 10855491 10.1007/s004120050408

[53] von der Haar B, Sperling K, Gregor D. Maturing *Xenopus* oocytes induce chromosome condensation in somatic plant nuclei. Exp Cell Res. 1981;134:477–81. 10.1016/0014-4827(81)90450-x. 6168480 10.1016/0014-4827(81)90450-x

[54] Wojtasz L, Daniel K, Roig I, Bolcun-Filas E, Xu H, Boonsanay V, Eckmann CR, Cooke HJ, Jasin M, Keeney S, McKay MJ, Toth A. Mouse HORMAD1 and HORMAD2, two conserved meiotic chromosomal proteins, are depleted from synapsed chromosome axes with the help of TRIP13 AAA-ATPase. PLoS Gene. 2009;5: e1000702. 10.1371/journal.pgen.1000702 PMC2758600 19851446

[55] Yost S, de Wolf B, Hanks S, Zachariou A, Marcozzi C, Clarke M, de Voer R, Etemad B, Uijttewaal E, Ramsay E, Wylie H, Elliott A, Picton S, Smith A, Smithson S, Seal S, Ruark E, Houge G, Pines J, Kops GJPL, Rahman N. Biallelic TRIP13 mutations predispose to Wilms tumor and chromosome missegregation. Nat Genet. 2017;49:1148–51. 10.1038/ng.3883. 28553959 10.1038/ng.3883 PMC5493194

[56] Zanders S, Alani E. The pch2Delta mutation in baker’s yeast alters meiotic crossover levels and confers a defect in crossover interference. PLoS Genet. 2009;5: e1000571. 10.1371/journal.pgen.1000571. 19629178 10.1371/journal.pgen.1000571 PMC2709914

[57] Zhang Z, Li B, Fu J, Li R, Diao F, Li C, Chen B, Du J, Zhou Z, Mu J, Yan Z, Wu L, Liu S, Wang W, Zhao L, Dong J, He L, Liang X, Kuang Y, Sun X, Sang Q, Wang L. Bi-allelic missense pathogenic variants in TRIP13 cause female infertility characterized by oocyte maturation arrest. Am J Hum Genet. 2020;107:15–23. 10.1016/j.ajhg.2020.05.001. 32473092 10.1016/j.ajhg.2020.05.001 PMC7332649

[58] Zhang X, Hu C, Wu L. Advances in the study of genetic factors and clinical interventions for fertilization failure. J Assist Reprod Genet. 2023;40:1787–805. 10.1007/s10815-023-02810-2. 37289376 10.1007/s10815-023-02810-2 PMC10371943

