# Supplementary figures and images for: A homozygous TRIP13 pathogenic variant associated with familiar oocyte arrest and prematurely condensed sperm chromosomes
Source: Mol Cytogenet. 2025 Jul 23;18:17. doi: 10.1186/s13039-025-00722-7 (PMC12285012; doi:10.1186/s13039-025-00722-7)

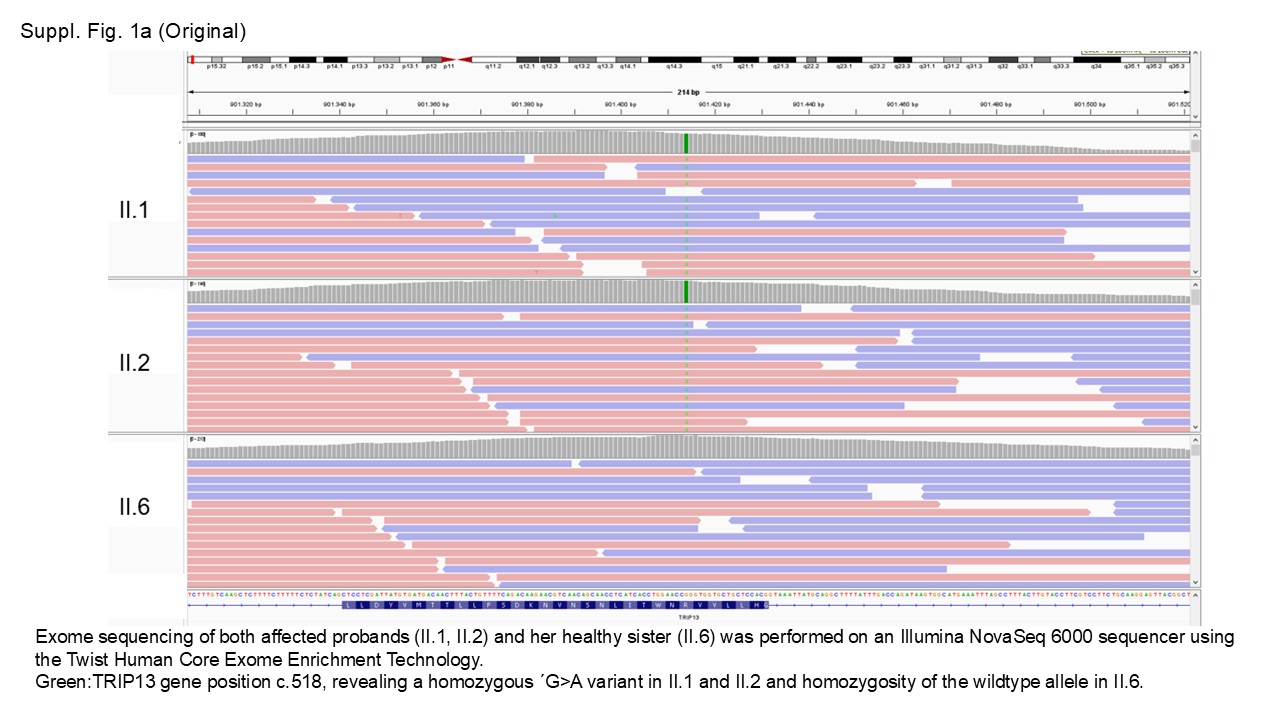

Supplement: Supplementary file 1 — Supplementary material 1 [file 13039_2025_722_MOESM1_ESM.jpg]

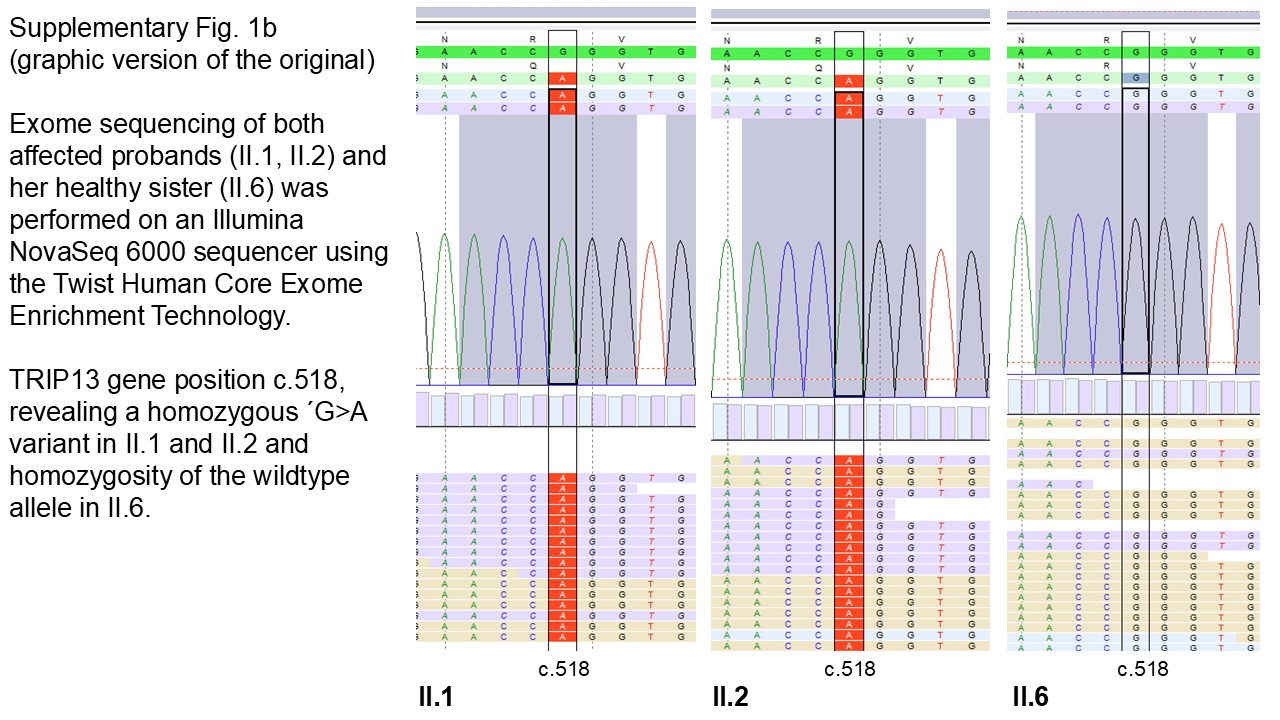

Supplement: Supplementary file 3 — Supplementary material 3 [file 13039_2025_722_MOESM3_ESM.jpg]

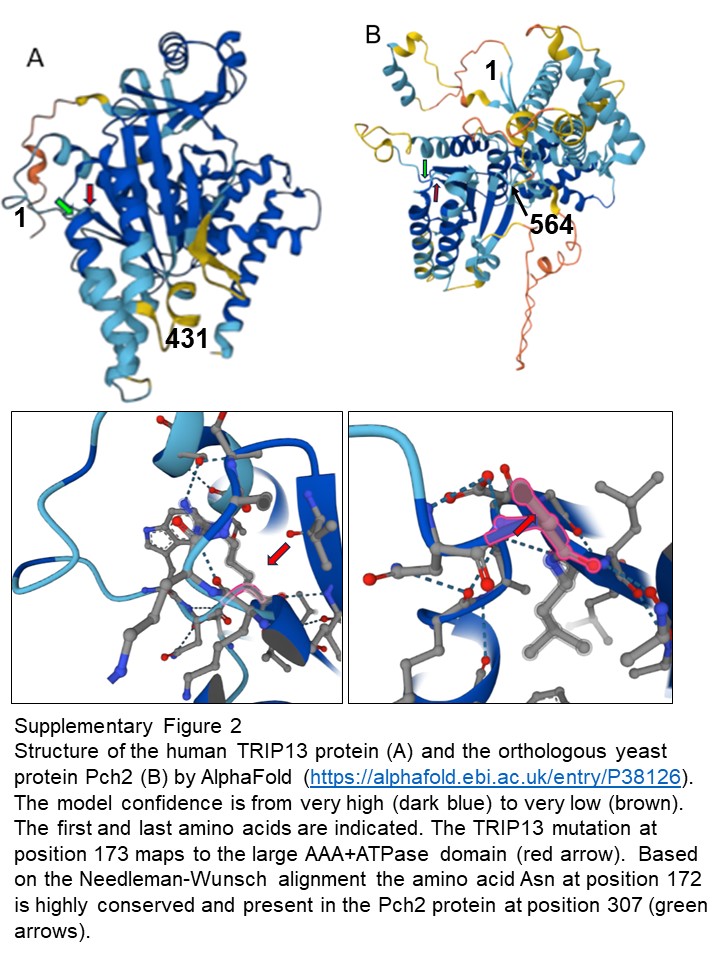

Supplement: Supplementary file 4 — Supplementary material 4 [file 13039_2025_722_MOESM4_ESM.jpg]
