# Supplementary material for: A homozygous TRIP13 pathogenic variant associated with familiar oocyte arrest and prematurely condensed sperm chromosomes
Source: Mol Cytogenet. 2025 Jul 23;18:17. doi: 10.1186/s13039-025-00722-7 (PMC12285012; doi:10.1186/s13039-025-00722-7)
Supplement: Supplementary file 2 — Supplementary material 2 [file 13039_2025_722_MOESM2_ESM.pdf]

Ms TRIP13: Supplementary Material

Supplementary Table 1: Comparison of SCE rates

proband	Cells analyzed	SCE rate	SCE/cell	P value #
II.1 affected	50	444	8.9	< 0.001
II.2 affected	50	241	4.8	> 0.5
I.1 heteroz.	49	244	5.0	> 0.5
II.5 heteroz.	51	228	4.5	> 0.5
II.3 heteroz.	51	302	5.9	> 0.5
control	50	246	4.9	-

comparison with the control (Mann-Whitney U-Test, two tailed)

Suppl. Table 2: Exome sequencing of both affected probands (II.1, II.2) and their healthy sister (II.6).

Exome sequencing was performed on an Illumina NovaSeq 6000 sequencer. 443 genes associated with infertility, familial cancer and increased SCE rate were evaluated. Variants were analyzed regarding their occurrence and frequencies in variant databases, their segregation and their consequences as well as regarding their plausibility in light of the phenotype (Databases: ClinVar, Gene Reviews, HPO, Med Gen, Omim).

II.1: affected sister with increased SCE-rate

1.NR5A1 Gene (nuclear receptor subfamily 5 group A member 1)

chr9:124493082C>A, exon 5: c.938G>A (p.Arg313His): pathogenic/likely pathogen

Disease name: 46,XX sex reversal 4 (OMIMP: 617480)

Comment: Only 20% of the reads carried this allele. Therefore, mosaicism as a result of a mutation in the lymphoblastoid cell line cannot be excluded. The two sisters examined were homozygous for the wild-type allele. An influence of this heterozygote variant on the SCE-rate is highly unlikely.

2.RNF212 ring finger protein 212 OMIMG 612041 ENTREZ ID 285498 ENSG00000178222

Homozygosity for a Deletion-Insertion variant in exon 4: c.720_721delinsGGCTGGCTCCAGCCTGGGCAG; p.R240_S241delinsGWLQPGQ

This allele was classified as “likely benign”.

Comment: RNF212 encodes a RING finger protein that is involved in meiotic recombination (Reynolds et al. 2013). A homozygous *RNF212* variant, c.111dupT, resulted in nonobstructive azoospermia (Riera-Escamilla et al. 2019). As this gene is involved in recombination processes, an influence of this variant on the SCE rate, though rather unlikely, cannot be ruled out in principle.

II.1: affected sister with normal SCE-rate

No phenotypically relevant variants detected, except TRIP13.

II.6 healthy sister

No phenotypically relevant variants detected.

Supplementary Table 3: Yeast complementation – comparison of P1/P2 and R1/R2 values

strain	Colo-nies	P1 lys$^+$ leu$^+$	P2 lys$^-$ leu$^-$	P value	Colo-nies	R1 lys$^+$ leu$^-$	R2 lys$^-$ leu$^+$	P value #
Y20000	158	71	87	0.43	58	26	32	0.71
TRIP13wt	186	113	73	0.047	131	64	67	1.0
TRIP13mut	498	305	193	0.0004	173	84	89	0.91

statistical analysis with Fisher´s exact test, two-tailed.

Y20000: wild type strain with all yeast genes, including *pch2*; TRIP13wt: deletion strain Y33326 with the *TRIP13* wild type gene; TRIP13mut: deletion strain Y33326 with the *TRIP13* mutant allele c.518G>A.

Supplementary Table 4: Statistical analysis of the recombination rate based on the TRIP13 spores with the highest original genotypes (in bold)

strain	P	R	P value #	Phenotype
Y20000	79	29	-	wild type
TRIP13wt	**113**	66	0.09	wild type
TRIP13mut	**305**	87	0.31	wild type

comparison with control strain (Y20000) with Fisher´s exact test, two tailed.
